# Comparison of a Plantain-Chicory Mixture with a Grass Permanent Sward on the Live Weight Gain and Meat Quality of Lambs

**DOI:** 10.3390/ani10122275

**Published:** 2020-12-02

**Authors:** Romina Rodríguez, Oscar Balocchi, Daniel Alomar, Rodrigo Morales

**Affiliations:** 1 Escuela de Graduados, Facultad de Ciencias Agrarias, Universidad Austral de Chile, Casilla 567, Valdivia 5090000, Chile; rominarodriguezmv@gmail.com; 2 Instituto Producción Animal, Facultad de Ciencias Agrarias, Universidad Austral de Chile, Casilla 567, Valdivia 5090000, Chile; obalocch@uach.cl (O.B.); dalomar@uach.cl (D.A.); 3 Instituto de Investigaciones Agropecuarias, INIA Remehue, Ruta 5 Norte km 8, P.O. Box 24–0, Osorno 5290000, Chile

**Keywords:** lamb meat, *Plantago lanceolata*, *Cichorium intybus*, carcass quality, sheep

## Abstract

**Simple Summary:**

In southern Chile, lamb production systems are based on the grazing of temperate swards. During late spring and early summer, grass-based swards can display herbage with suboptimal growth and quality, reducing lamb production. Plantain and chicory are alternative herbages that improve animal performance, especially in periods with a risk of water deficits. This study was carried out during late spring, to evaluate live weight gain and meat quality in lambs grazing on plantain-chicory sward or grass-based permanent sward. The results show that lambs fed with herbs (plantain-chicory) have a similar final live weight, carcass weight and meat quality to those fed with grass. Plantain and chicory swards are good form of alternative herbage for finishing lambs in late spring to maintain the performance of the animals.

**Abstract:**

Under the predicted conditions of climate change, the productivity of temperate grasslands may be affected by drought stress, especially in spring and summer. In this scenario, water-deficit-tolerant species such as plantain and chicory are interesting alternatives for use in sheep production systems. In this study, we compared a mixture of plantain and chicory herbage (PCH) with a grass-based permanent sward (GBS) on the weight gain and meat quality of lambs finished on these grasslands. Fifteen weaned lambs (31.3 kg and 4 months of age) were assigned to each treatment for seven weeks in late spring and live weight gain (LWG), carcass and meat quality were evaluated. There was a tendency (*p* = 0.09) in final weight (40.3 ± 0.8 kg) and live weight gain (173 ± 10 g/d) to be higher in PCH compared to GBS. Carcass weight, dressing percentage and meat quality in terms of pH, color and tenderness did not differ (*p* > 0.05) and were considered to be of good quality. We concluded that both swards result in comparable lamb performance and good meat quality.

## 1. Introduction

Consumers are increasingly recognizing the value of healthier animal products from sustainable production systems with a low impact on the environment [1]. At the same time, climate change is affecting rainfall patterns and increasing temperatures in temperate humid regions, causing water deficit problems, with an impact on the yield of permanent grasslands dominated by perennial ryegrass, other grasses and white clover, mainly in late spring and early summer [2], which is the time when lambs are normally finished in sheep production systems based on pastures.

The use of alternative forage species for sheep, such as *Plantago lanceolata* (plantain) and *Cichorium intybus* (chicory) is interesting, since these species can withstand the above limitations and maintain growth and a high nutritional quality, which is important for growing animals [3,4,5,6]. Furthermore, these species have a high mineral content, anti-inflammatory effects and an anthelmintic potential [3,4,5].

It has been reported that lambs finished on these species can achieve weight gains exceeding 200 g/d, higher than on grass-based swards, especially in the summer season [4,7]. The use of mixtures of herbs and legumes in spring and autumn could improve the performance of lambs, achieving live weight gains up to 250 g/d post-weaning [8,9]. The importance of achieving high lamb weights and being finished on pastures leads to a higher acceptability on the part of consumers, who seek healthier meats from pasture-fed animals raised in the open, conditions normally associated with superior animal welfare [10,11].

Animal foods, especially red meat, are considered a rich source of bioactive nutrients and antioxidants, containing high quality proteins, vitamins and high levels of essential minerals such as iron and zinc [12]. Some meat characteristics are key factors in consumers’ decisions at the moment of purchase. For instance, the color must be adequate to show that the meat is of high quality and natural [13], and the same applies to flavor and tenderness. Studies concerning the use of a plantain-chicory mix on live weight gain in lambs are scarce, because the reports usually include red clover in the mix or use monocultures of chicory or plantain.

In the present study, we compared a mixture of plantain and chicory with a permanent pasture mainly composed of temperate grasses on the productive performance, carcass characteristics and meat quality of weaned lambs. Our hypothesis was that a sward composed of plantain and chicory could improve the productive parameters and meat quality compared to a grass-based permanent sward.

## 2. Materials and Methods

The experiment was conducted at the Sheep Unit of the Austral Agricultural Experimental Station of Universidad Austral de Chile (UACH) in Valdivia (39°43′ S; 73°14′ W) Chile for 49 days in late spring (November–December). The use of animals and their management procedures for this study was approved by the Bioethics Committee of UACH.

This study was derived from the same swards and animals already published in Rodríguez et al. [14] and Rodríguez et al. [15].

### 2.1. Forage Treatments

Two different pastures in their third year of establishment were tested as treatments—a mixture of plantain and chicory (PCH) and a grass-based permanent sward (GBS). The total grazing area was 9000 m^2^. This was divided six paddocks (three per treatment), each of 1500 m^2^. Details of establishment and management of pastures can be found in Rodríguez et al. [14]. Concisely, the PCH sward was sown with 8.4 kg/ha plantain (*Plantago lanceolata* cv. Ceres Tonic) and 8.4 kg/ha chicory (*Cichorium intybus* cv. Choice). The GBS sward contained perennial ryegrass (*Lolium perenne* cv. Rohan, 8.6 kg/ha), brome (*Bromus valdivianus* cv. Póker, 11.52 kg/ha), tall fescue (*Festuca arundinacea* cv. Kora, 8.64 kg/ha), cocksfoot (*Dactylis glomerata* cv. Safin, 5.76 kg/ha), Yorkshire fog (*Holcus lanatus*, (2.88 kg/ha) and white clover (*Trifolium repens*, cv. Weka, 3.6 kg/ha). Swards at sowing (March) were fertilized with N (30 kg/ha), P (120 kg/ha), K_2_O (60 kg/ha) and limestone (CaCO_3_, 2 ton/ha). In October and November, N was applied to complete 100 kg/ha. The same amounts of N, P and K were applied in the second and third years.

### 2.2. Animal Managements

Thirty single male Austral (a local breed developed from a Finn × Romney cross) lambs (average live weight of 31.3 ± 2.4 kg and approximately four months of age) were randomly distributed at weaning to the PCH and GBS treatments, three paddocks per treatment, five lambs per paddock. A pre-experimental period of 1 week was applied for the lambs to adjust to the different herbage types (grazing both swards) and to familiarize them with handling and weighing. Animals were strip-grazed using electric fencing. Grazing time was from 08:00 to 20:00 and lambs were housed overnight for security reasons (predation by stray dogs). Rotation length depended on herbage growth and a minimum post-grazing surface height of 5 cm for GBS and 7 cm for PCH were used [8]. Drinking water was available ad libitum.

### 2.3. Forage Measurements

Five pre-grazing and post-grazing circular areas (0.25 m^2^) at ground level were measured by clipping to determine herbage dry matter (DM) from each strip at the time lambs were moved. These samples were oven dried at 60 °C for 48 h and weighed to determine DM. Apparent DM intake was determined by the difference between pre- and post-grazing DM mass. For forage quality, samples were taken from each paddock at 4 cm height (mimicking lamb intake behavior). Four samples were obtained from each strip, pooled per paddock and frozen until being analyzed for dry matter (MS), crude protein (CP) and total ash, according to Association of Official Agricultural Chemist (AOAC), [16] (methods 930.15, 990.03 and 942.05, respectively), as well as neutral detergent fiber (NDF) [17], acid detergent fiber (ADF) [16] (method 973.18) and metabolizable energy (ME), estimated with a local regression [18] on the digestible organic matter content (DOMD) obtained in vitro using the two-stage rumen fluid-pepsin method. The minerals calcium (Ca), magnesium (Mg), potassium (K), sodium (Na), iron (Fe), copper (Cu), zinc (Zn) and manganese (Mn) were analyzed according to AOAC (method 975.03) [16]. The methodology for phosphorous (P) was from AOAC (method 22) [19]. Botanical composition was evaluated by cutting 10 herbage samples to ground level from each sward. A subsample was taken to determine the percentage of each species and dead matter present in the treatments before each grazing event. Samples were separated into chicory, plantain, white clover, perennial ryegrass, cocksfoot, tall fescue, Yorkshire fog, brome and dead matter. Fractions were dried at 60 °C for 48 h and weighed to report botanical composition on a DM basis.

### 2.4. Animals and Meat Measurements

Lambs were weighed weekly (two days consecutively) at turnout in the morning, to determine live weight and live weight gain (LWG). After seven weeks they were sent to a commercial abattoir (Mafrisur^®^, Osorno, Chile). Hot (HCW) and cold (CCW) carcass weight were recorded after storage at 4 ± 2 °C for 48 h. Dressing out percentage (DO%) was calculated on hot carcass weight and pre-slaughter weight. Each carcass was split down the vertebrae into two bilateral halves. From the right half-carcass, the longissimus thoracis et lumborum (LTL) was removed (10th to 12th rib). The pH was measured in the LTL muscle by means of a pH meter with a penetrating electrode (Hanna 99163; Hanna Instruments, Woonsocket, RI, USA).

The LTL was cut into steaks (3 cm thick) that were kept at room temperature for 30 min. Steaks were used to determine meat color with three measurements using the Commission Internationale de l’Eclairage (CIELAB) system (where L* measures relative lightness, a* relative redness, b* relative yellowness) with a colorimeter (CR-400, Konica Minolta Inc., Tokyo, Japan).

Samples were cooked without external fat in an electric oven (EKA^®^, KF 620 model, Famava, Santiago, Chile) at 170 °C until they reached a central temperature of 70 °C, monitored through a digital thermometer-type thermocouple (Sper Scientific Ltd., model 800024, Scottsdale, AZ, US). Samples were then chilled at 4 ± 2 °C for 24 h. Subsequently, six to ten subsamples of 1.3 cm diameter were extracted and shear force was measured with a Texture Analyser (Stable Micro Systems, PLUS-UPGRADE model) using the Warner-Bratzler (WBSF) method. Meat samples (50 g) were analyzed for ether extract [20] (920.39 method) and minerals (P, Ca, K, Zn, Cu, Fe and Mn) using the AOAC [19] methodology (method 22).

### 2.5. Statistical Analysis

The experimental design was a randomized complete block design. Herbage quality and carcass and meat quality variables were analyzed by ANOVA with the sward type as a fixed effect using the general linear model. Live weights and LWG of lambs and apparent feed intake data were analyzed using a repeated measures mixed model, with week as the repeated factor. Proc GLM (General linear models) and MIXED procedures (SAS Version 9.2, SAS Institute Inc., Cary, NC, USA) were used respectively.

## 3. Results

### 3.1. Herbage Quality

The GBS had 32% perennial ryegrass, followed by cocksfoot (23%), brome (13%), white clover (9%), tall fescue (2%) and Yorkshire fog (2%). The PCH mixture was represented mainly by chicory (35%) and plantain (33%), although white clover presented a 24% contribution to dry matter and 2% were classified as other species. The dead matter was higher in GBS, at 19%, whereas PCH had only a 6% contribution.

The nutritional quality of herbage is shown in Table 1. A higher content of ash was observed for PCH (*p* < 0.05) compared to GBS forage. The crude protein did not differ between swards (*p* > 0.05), averaging above 200 g/kg. The ME and fiber fractions differed, the former being higher in GBS (*p* < 0.05). Both ADF and NDF were lower in PCH. The macro-minerals Ca and Na were two and three times higher in PCH (*p* < 0.05) than in GBS. Phosphorus was also higher in PCH, whereas K and Mg were not different between treatments (*p* > 0.05). Trace elements Cu and Zn showed a tendency (*p* = 0.08) to be higher for PCH. Iron and Mn did not differ between treatments (*p* > 0.05).

### 3.2. Animal Performance

**Figure 1:**
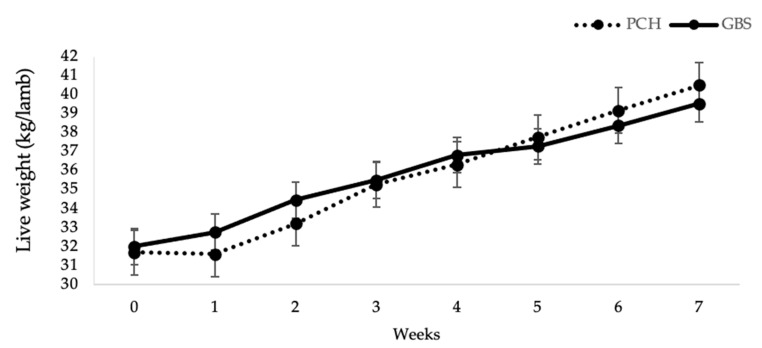
Weekly weights in lambs grazing the plantain-chicory mixture (PCH) and the grass-based permanent sward (GBS).

Weekly and final body weights of lambs did not differ between swards and no interaction (*p* > 0.05) was found with weeks (Figure 1).

**Figure 2:**
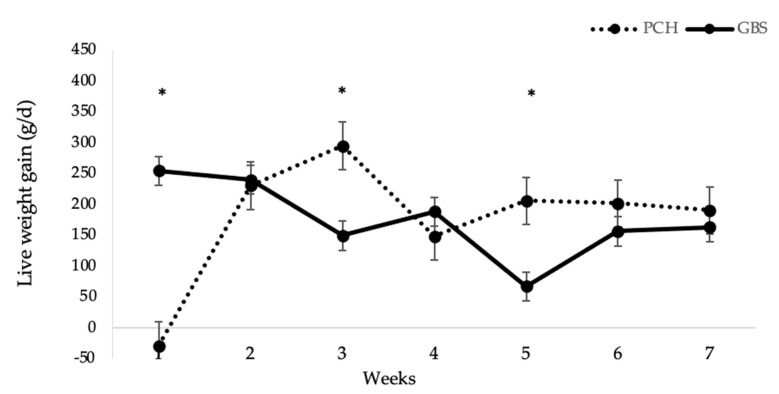
Live weight gain of lambs grazing on plantain-chicory (PCH) and grass-based permanent sward (GBS). * *p* < 0.05.

Similar average LWG values were found between swards, but a significant interaction (*p* < 0.05) was detected with week (Figure 2), with a slight weight loss in the first week and a higher gain (*p* < 0.05) in the third and fifth weeks for PCH, compared to GBS.

Overall performance (Table 2) did not show differences (*p* > 0.05) in initial LW and apparent DM intake in lambs from both treatments, although PCH lambs had a tendency (*p* = 0.09) to present a higher final LW and LWG than GBS lambs.

### 3.3. Carcass and Meat Quality

Carcass and meat quality variables are presented in Table 3. No differences (*p* > 0.05) were found between PCH and GBS treatments in hot (HCW) or cold (CCW) carcass weight.

Dressing out percentages did not differ (*p* > 0.05) and were above 40%. Muscle pH values were lower (*p* < 0.05) for PCH than GBS and meat quality, and in terms of shear force and color (lightness L*, redness a*, yellowness b*) the values were similar (*p* > 0.05). The chemical composition of meat did not differ (*p* > 0.05) between treatments in terms of intramuscular fat and minerals.

## 4. Discussion

Our hypothesis stated that finishing lambs on a sward composed of plantain and chicory would result in improved productive parameters and meat quality, compared to those finished on a grass-based permanent sward. This assumption is based on factors such as a high nutritional quality of these broad leaved species, an adequate DM intake and an efficient utilization of these nutrients. Nutritional quality differed between treatments, with the exception of crude protein. Total ash was higher in PCH, but NDF was lower compared to GBS. Previous reports have shown that, compared with perennial ryegrass, plantain and chicory contain higher levels of ash [4,5] and that plantain contains higher concentrations of Se, Cu, Mg, Na, Cl, Fe, Co, Ca and S [21], whereas chicory contains higher concentrations of Na, Ca, K and Mg [4]. Box et al. [22] found that Na and Ca were two and three times greater in plantain compared to perennial rye grass-white clover pasture. The ME content was higher in GBS compared to PCH, agreeing with Sube et al. [23] and both energy and protein content in PCH and GBS were adequate, considering the nutritional requirements recommended by the National Research Council (NRC) [24] for growing-finishing lambs. The low NDF in the herb sward could be explained by the fact that chicory leaves have lower cellulose and hemicellulose concentrations and higher soluble sugars and pectin than perennial ryegrass [25]. Plantain leaves, on the other hand, have lower NDF, especially cellulose, although they have more lignin in the cell wall [4,5,21].

The average LWG values of lambs for both treatments were lower than those reported in other studies (over 250 g/d) in the same season in New Zealand [8], although PCH showed a non-significant slight advantage over GBS. An insufficient voluntary intake could have been a factor explaining the limited LWG obtained in the present work. To achieve more than 200 g/d, [24] proposes a DM intake of 1.3 kg/d, higher than the estimated intake in our study. Although the post-grazing residues set for this trial were planned to allow for ad-libitum intake, it should be kept in mind that lambs were housed overnight to prevent the risk of animal theft and predation by feral dogs, and this procedure probably reduced effective grazing time. Moreover, anthelmintic treatment was not applied in the post-weaning period. Parasite burden could be a relevant factor in the resulting LWG of lambs and the post-weaning use of an anthelmintic could be effective in reducing gastrointestinal nematodes [26].

The live weight gain in the first week was higher for the lambs on the GBS sward, but remained similar or lower thereafter, compared to PCH lambs. Although chicory and plantain have a high nutritional value compared to several temperate grasses, especially in late spring, these herb species present a lower palatability (by secondary metabolites) than perennial ryegrass and white clover [4,27]. The recommendation is to associate it with other species, mainly in the summer and autumn season [28,29]. These herbs do not limit the intake of the animals [7,9,21,30] if an acclimation or adaptation period is provided, which is convenient due to the presence of some bioactive compounds that can generate a bitter taste.

Final LW tended to be higher for PCH lambs, at 2 kg per head on average, equivalent to about two extra lambs per hectare for the herb treatment. This could be explained by the good quality of the forage, and even though GBS presented a greater ME (metabolizable energy) content, this value is obtained by an in vitro method and does not necessarily represent the actual in vivo utilization of nutrients. It is likely that the high NDF content of GBS probably resulted in a lower rumen outflow rate, explaining the slightly lower intake estimated for this sward [9].

Carcass parameters were similar between treatments, but a tendency was found toward higher carcass weights (HCW and CCW) in PCH, consistent with the tendency toward a higher final LW in this treatment. The HCW, at more than 16 kg, is somewhat superior to that described by Golding et al. [9], but lower than those reported in other studies [8,25,31]. The dressing-out percentage exceeded 40%, which is within the range of values obtained in the above-mentioned studies. Higher carcass weights and DO percentages have been obtained with mixtures of chicory and plantain, compared to perennial ryegrass and white clover. This could be attributed to the high nutritional quality of plantain and chicory, but also to the fact that lambs grazing on herbs had a greater weight at slaughter compared to lambs fed on grass-clover treatments [32].

Meat quality parameters are fundamental at the time of choosing a particular meat by consumers. The meat quality parameters in both treatments did not differ. Although the pH in PCH meat was lower than in GBS meat, both are within the range expected to achieve a desirable meat quality and agree with other studies that report values between 5.5 to 5.8 [13]. However, pH values between 5.4 and 5.6 result in meats of better flavor, color and tenderness. It is also an indicator of the progression of proteolysis and could influence the physical and chemical characteristics of meat that are related with tenderness (shear force) of meat [33]. The values obtained for shear force in our work are indicative of a tender meat and fall within the lower limits described in different studies [31,34,35] that have used different breeds and a range of grazing swards. This improved tenderness could result from the fact that some types of fibers are more susceptible to proteolytic degradation [36], although the type of fibers was not evaluated in this work.

Color is an important trait for consumers when selecting a meat at the time of purchase, as this can be associated with its condition (good or bad) and freshness [13]. The meat color results of our study were similar to those observed by other authors [31,34,35]. Schreurs et al. [37] recorded similar color values for loin samples from lambs grazing on perennial ryegrass-white clover pasture, plantain-clover mixes and chicory-plantain-clover mixtures. Khliji et al. [38] reported in Australia that an acceptable meat has L* and a* values higher than 34 and 9.5, respectively. Therefore, our results denote a meat that could be categorized as acceptable by consumers.

The IMF content did not differ between treatments and was lower than that suggested for a good eating quality (above 4%) [39]. The animals finished on pasture tend to show lower IMF content than those finished on grain [40]. This content was close to that reported by De Brito et al. [35] in a study based on grazing. This lamb meat can be considered a lean meat and is suitable to enter the health food market [13,40].

The meat’s mineral content was similar for both treatments. Lamb meat is an important source of iron and zinc, with a high bioavailability compared to those found in plants [41,42]. Meat can contribute 50% of the recommended daily amount of iron in humans [12].

## 5. Conclusions

Although our hypothesis was not supported by the results obtained, the mixture of broad-leaved herbs had a tendency to improve weight gains and carcass weight. Meat quality did not differ between treatments, with a good tenderness and color. Further studies should be done to compare and to corroborate the findings reported in the present manuscript, e.g., evaluating the effect of pure species and cultivars, studying the effect of herbs over a longer period and/or performing the experiment in dry summer conditions.

## Figures and Tables

**Table 1:** Herbage quality and mineral content in the plantain-chicory mixture (PCH) compared to the grass-based sward (GBS).

Minerals	PCH	GBS	SEM	*p*-Value
Ash (g/kg)	111.7	86.1	3.69	<0.001
CP (g/kg)	218.2	196.7	10.39	0.333
ME (MJ/kg)	11.1	11.7	0.12	0.008
NDFom (g/kg)	257.8	443.3	11.91	<0.001
ADFom (g/kg)	205.9	263.8	19.44	0.052
Ca (g/kg)	11.7	6.0	1.30	0.020
Na (g/kg)	5.3	1.8	0.80	0.007
P (g/kg)	3.4	2.6	0.20	0.051
K (g/kg)	33.6	28.0	2.30	0.261
Mg (g/kg)	1.6	1.8	0.20	0.595
Cu (mg/kg)	18.6	14.1	1.30	0.085
Fe (mg/kg)	162.1	153.3	20.40	0.614
Mn (mg/kg)	93.7	108.4	11.80	0.675
Zn (mg/kg)	30.0	23.6	2.15	0.083

CP: crude protein; ME: metabolizable energy; NDFom: ash-free neutral detergent fiber; ADFom: ash-free acid detergent fiber; SP: soluble protein; SEM: standard error of the mean.

**Table 2:** Effect of sward treatment (plantain-chicory and grass-based sward) on animal performance parameters.

Animal Perfomance	PCH	GBS	SEM	*p*-Value
LWG (kg/d)	0.197	0.149	0.01	0.090
Initial LW	31.7	32.0	0.58	0.706
Final LW (kg)	41.4	39.3	0.85	0.098
Apparent intake (kg DM/lamb/day)	1.1	1.0	0.07	0.270

PCH: Plantain-chicory sward; GBS: grass-based permanent sward; LWG: live weight gain; LW: live weight; SEM: standard error of the mean; DM: dry matter.

**Table 3:** Effect of swards on carcass parameters and meat quality characteristics in lambs.

Meat Parameters	PCH	GBS	SEM	*p*-Value
HCW (kg)	17.3	16.3	0.33	0.138
CCW (kg)	16.8	15.8	0.33	0.130
DO (%)	41.8	41.4	0.58	0.756
pH	5.5	5.6	0.02	0.015
Shear force (N)	15.1	16.1	0.49	0.417
L *	41.8	41.3	0.28	0.412
a *	19.9	20.3	0.24	0.426
b *	8.3	8.2	0.19	0.797
IMF (%)	1.1	1.2	0.07	0.706
P (%)	0.2	0.2	0.001	0.111
Ca (%)	0.009	0.007	0.001	0.459
K (%)	0.3	0.3	0.003	0.935
Zn (mg/kg)	17.8	18.5	0.79	0.540
Cu (mg/kg)	1.5	1.6	0.07	0.631
Fe (mg/kg)	11.7	12.4	0.34	0.198
Mn (mg/kg)	0.2	0.1	0.016	0.204

PCH: plantain-chicory; GBS: grass-based sward; HCW: hot carcass weight; CCW: cold carcass weight; DO: dressing out percentages; L*: lightness; a*: redness; b*: yellowness; IMF: intramuscular fat; SEM: standard error of the mean.
